# Master Athletes Are Extending the Limits of Human Endurance

**DOI:** 10.3389/fphys.2016.00613

**Published:** 2016-12-12

**Authors:** Romuald Lepers, Paul J. Stapley

**Affiliations:** ^1^Cognition Action et Plasticité Sensorimotrice UMR 1093, Institut National de la Santé et de la Recherche Médicale (INSERM), University of Bourgogne Franche-ComtéDijon, France; ^2^Neural Control of Movement Laboratory, Faculty of Science, School of Medicine, Medicine and Health, University of WollongongWollongong, NSW, Australia

**Keywords:** aging, performance, masters, marathon, triathlon, swimming, ultra-endurance, cycling

## Abstract

The increased participation of master athletes (i.e., >40 years old) in endurance and ultra-endurance events (>6 h duration) over the past few decades has been accompanied by an improvement in their performances at a much faster rate than their younger counterparts. Aging does however result in a decrease in overall endurance performance. Such age-related declines in performance depend upon the modes of locomotion, event duration, and gender of the participant. For example, smaller age-related declines in cycling performance than in running and swimming have been documented. The relative stability of gender differences observed across the ages suggests that the age-related declines in physiological function did not differ between males and females. Among the main physiological determinants of endurance performance, the maximal oxygen consumption (VO_2max_) appears to be the parameter that is most altered by age. Exercise economy and the exercise intensity at which a high fraction of VO_2max_ can be sustained (i.e., lactate threshold), seem to decline to a lesser extent with advancing age. The ability to maintain a high exercise-training stimulus with advancing age is emerging as the single most important means of limiting the rate of decline in endurance performance. By constantly extending the limits of (ultra)-endurance, master athletes therefore represent an important insight into the ability of humans to maintain physical performance and physiological function with advancing age.

## Increase in participation of master athletes in (ultra)-endurance events

Over the past three decades, there has been a continual increase in the number of master athletes (i.e., >40 years old) in endurance and ultra-endurance (>6 h) events (Zaryski and Smith, 2005). Indeed, the percentage of male finishers older than 40 years is frequently higher than those under 40 years. At the New-York marathon, male master runners represent now more 50% of total male finishers while female master athletes represented 40% of total female finishers, respectively (Lepers and Cattagni, 2012). This finding corroborates previous observations for 100- and 161-km ultra-marathon running where master runners represent the greatest part of the finishers; up to 73% for 100-km (Hoffman et al., 2010; Knechtle et al., 2012). Similar trends have been observed for multiple discipline events such as triathlon (swimming, cycling, running). For example, master triathletes represent now more than 55% of the total field for males and more than 45% of the total field for females at the World championship Ironman triathlon in Hawaii (Lepers et al., 2013a). The increase in participation of master athletes in endurance events inevitably has an influence on the mean age of the finishers. Generally finishers are older than 40 years in endurance and ultra-endurance events, e.g., 47 years for 100-km running (Knechtle et al., 2012), 43–44 years for 161-km running (Hoffman et al., 2010), or 43 years for ultra-cycling such as Race Across America (Shoak et al., 2013).

Different factors could explain the increase in participation of master athletes in endurance events. With the increase in life expectancy and training facilities, such as specific masters programs, the increased participation of oldest athletes (>60 years old) described may be a reflection that these athletes, who may in some cases be retired, have more available time and resources at their disposal to train and therefore to compete (Reaburn and Dascombe, 2008). However, sporting background, social category and working profession of the master athletes all remain unknown. Some may have the experience of many years of training and competition, while others only begin as they approach middle-age and beyond.

The relative increase in participation of master athletes in endurance events could also result from a decrease in participation in the younger age groups. Younger people wanting to try endurance activities may be more and more attracted to more “fun” events, such as “Obstacle course races” or “Mud runs” (http://www.outsideonline.com/1892576/american-gladiators). Indeed, an estimated 4.5 million participated in such races in the USA in 2015 alone (http://www.usatoday.com/story/sports/2015/11/02/obstacle-races-going-mainstream-more-popular-than-marathons/73743474/).

## Elite ultra-endurance athletes get older

The current age of elite marathoners is around 30 years for both males and females (Hunter et al., 2011), but the age of peak performance in endurance events generally increases as race distance increases (Knechtle et al., 2014; Romer et al., 2014). Interestingly, data from ultra-endurance events reported that the age of the elite athletes has increased over the past few decades as have their performances. For example, at the Hawaii Ironman triathlon, since the 80's, the age of the annual top 10 finishers increased over time from 26 to 35 years for females and from 27 to 34 years for males, respectively (Gallmann et al., 2014). Anecdotally, this year (2016) the winner of the New-Zealand Ironman triathlon (i.e., Cameron Brown) was 43 years and became the oldest winner of any Ironman race. Similarly, the winner of the Austria Ironman triathlon (i.e., Marino Vanhoenacker) was 40 years old and the second placed athlete (Viktor Zyemtsev), 43 years old. Similar trends have been observed for elite ultra-marathoners who also became older over time (Hoffman and Wegelin, 2009; Eichenberger et al., 2012). During the past four decades, the age of the top 5 runners at the “Western States 161-km Endurance Run” increased from the early to the upper thirties (Hoffman and Wegelin, 2009). These data show that the upper age limit of elite ultra-endurance athletes has increased during the past decades bringing into question what the upper limit of the age of peak performance in elite ultra-endurance performance actually is.

## Performances of master athletes improved at a faster rate than in young athletes

**Figure 1:**
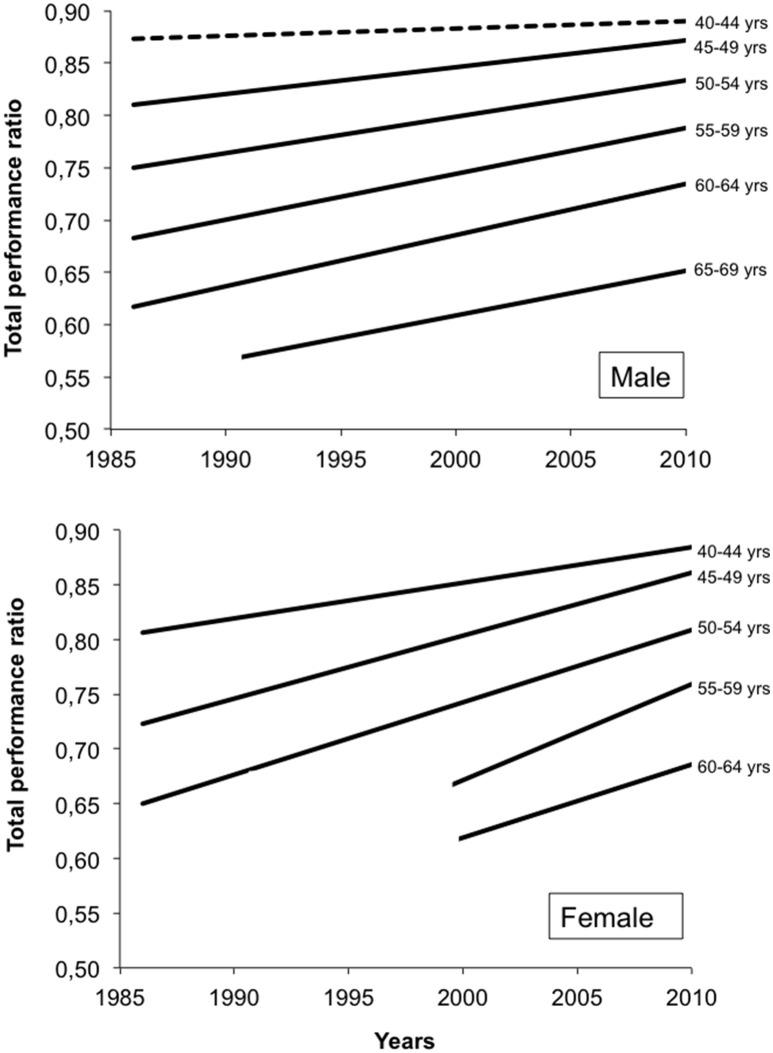
**Changes in performance ratio for total time at the Hawaii Ironman triathlon across the years for best male and female master triathletes**. A performance ratio equal to 1 corresponds to the performance of the top10 elite triathletes (<40 years old). Solid lines represent conditions where the slopes of the linear regressions were significantly different (*P* < 0.01) from zero. Dashed lines indicate the slopes of the linear regressions were not significantly different from zero. Lines cross only the range of years for which data were considered.

Age-related declines in endurance and ultra-endurance performance have been well described in the literature for swimming (Tanaka and Seals, 2003; Zamparo et al., 2012; Ferreira et al., 2016; Knechtle et al., 2016), cycling (Balmer et al., 2008; Baker and Tang, 2010; Capelli et al., 2016), running (Leyk et al., 2007; Hoffman, 2008; Hoffman et al., 2010; Knechtle et al., 2012; Lepers and Cattagni, 2012), and triathlon (Baker and Tang, 2010; Bernard et al., 2010; Lepers and Maffiuletti, 2011; Stevenson et al., 2013, Lepers et al., 2013b). The age-related decline in (ultra)-endurance performance with advancing age depends on the discipline and on gender. It ranges from 8% per decade (e.g., in 100-km male ultra-marathon runners; Knechtle et al., 2012) to 15% per decade (e.g., in Ironman female triathletes, Lepers et al., 2013a). Interestingly, the increase in participation of master athletes in ultra-endurance and endurance events over the past few decades has been accompanied by an improvement in their performance at a faster rate than young athletes. This has been observed for marathon running (Lepers and Cattagni, 2012) and also for triathlon (Lepers et al., 2013a,b; Stiefel et al., 2014). The improvement in performance of master athletes is more pronounced for the oldest age group categories (>60 years). For example, it has been shown that the best marathon male runners under 60 years did not significantly improve their performances at the New-York marathon during the 1980–2009 period, while average running time within the 70–74 years age group decreased by 7% over the same three decades (Lepers and Cattagni, 2012). Master female runners followed the same trends but the improvements occurred in all age group categories >50 years and were greater than for males. For a newest ultra-endurance sport such as Ironman triathlon that started in the early 80's (Lepers, 2008), the improvement in performance of master triathletes is much more pronounced compared to running. For example, at the Hawaii Ironman triathlon, the male 60–64 year age group triathletes improved their performance by 20% during 1986–2010 the period. The same rate of improvement was found for the best female 50–44 year age group triathletes (Lepers et al., 2013a; Figure 1).

The better physical condition of older athletes is likely to have increased participation, competitive spirit and performance (Maharam et al., 1999; Ransdell et al., 2009). The higher participation rates of master athletes increases the possibility of them achieving better results due to the competitive nature of the sport (Hunter and Stevens, 2013). Other reasons may explain the improved endurance performance of master athletes such as improvement of training facilities, new training methods (e.g., concurrent strength and endurance training, polarized training), master athlete coaching, nutritional strategies, and equipment (Reaburn and Dascombe, 2008; Louis et al., 2012; Piacentini et al., 2013; Brisswalter and Louis, 2014; Pugliese et al., 2015).

## Age-related decline occurs least in cycling

The age-related decline in endurance performance depends on the modes of locomotion. Studies on triathlon involving running, cycling, and swimming have offered the possibility of comparing age-related declines in three disciplines for the same athlete. Interestingly, it has been observed that there was a smaller age-related decline in cycling performance than in running and swimming performances for both short- and long-distance triathlons (Bernard et al., 2010; Lepers et al., 2010, 2016; Lepers and Stapley, 2011). These findings show that age-related declines in endurance performance are specific to the mode of locomotion, although the cause for such mode-specificity is not really clear. Several hypotheses have been proposed to explain the smaller declines in cycling performance with advanced age such as difference in mechanical power between in cycling and running, a lesser reduction in lactate threshold or economy during cycling, a greater muscle fatigue during running with age or greater training volume in cycling compared to running to limit the traumatic injuries (Easthope et al., 2010; Lepers et al., 2010, 2016), but these assumptions remain speculative and require further investigations. In addition, the age-related declines in ultra-endurance performance seem also depend on exercise duration at least for cycling and running. Indeed, it has been shown that the magnitude of the declines in cycling and running performances with advancing age during short distance triathlon (3 h) are less pronounced than during long distance triathlon (10 h), while the age-related decline in swimming performance are not influenced by triathlon duration (Lepers et al., 2010).

## Gender differences in performance have increased with age but may soon attenuate

Gender differences in endurance performance for elite athletes is generally close to 10% (Cheuvront et al., 2005) except for ultra-endurance swimming where females tend to reduce the gap with males (Lepers and Maffiuletti, 2011; Eichenberger et al., 2012; Knechtle et al., 2014). Men have a greater VO_2max_ than women because they have larger hearts, greater hemoglobin concentration, less body fat, and greater muscle mass per unit of body weight (Cheuvront et al., 2005). The other two primary factors that limit endurance performance at least for running including running economy and the “lactate threshold” seem not to differ between men and women (Joyner and Coyle, 2008). Gender differences in endurance performance are of biological origin and the gap between elite males and females is unlikely to narrow naturally. Several studies have shown that the gender difference in endurance performance increases with advanced age. It has been observed for running (Hunter and Stevens, 2013; Senefeld et al., 2016) and triathlon (Lepers and Maffiuletti, 2011; Lepers et al., 2013b) but is still controversial for swimming (Knechtle et al., 2016; Senefeld et al., 2016). Differences in swimming with advanced age appear to be less than in marathon running and may be related to a greater more depth in women's swimming than marathon running (Senefeld et al., 2016). The lower participation rate and less depth among women competitors in master age group categories is likely to amplify the difference between genders in endurance performance above that due to physiological differences alone (Hunter and Stevens, 2013). Environmental and social conditions and achievement motivation have played a role in limiting women's participation but it seems less and less true more recently (Deaner, 2013). For example, at the New-York marathon, differences between men and women in running times decreased for older age groups during the last three decades but since 2000 the differences have not significantly differed across age groups (Lepers and Cattagni, 2012). This relative stability of gender differences observed across the ages suggest that the age-related declines in physiological function did not differ between males and females. We suppose that in the near future, gender differences in endurance performance will be the same for all age group categories at least for the best older athletes, but this assumption will need to be verified.

## Age-related changes in physiological determinants of endurance exercise performance

Previous data indicate that decrease in maximal oxygen consumption (i.e., VO_2max_) is the predominant contributor to the decline in performance with advancing age (for details see Tanaka and Seals, 2008). Reductions in the ability to sustain a high fraction of VO_2max_, evaluated by blood lactate threshold and submaximal exercise economy may also contribute.

VO_2max_ declines by ~10% per decade after 30 years in healthy sedentary adults. It has been suggested that the rate of decline in VO_2max_ with age was smaller in master endurance athletes than in sedentary adults (Heath et al., 1981), however, this observation has been brought into question by more recent data. Indeed, some studies found that master endurance athletes demonstrate similar and even greater absolute rates of decline in VO_2max_ with age than healthy sedentary adults, as a result of greater baseline VO_2max_ as young adults and greater reductions in training with advancing age compared with sedentary adults (Fitzgerald et al., 1997; Eskurza et al., 2002). Trappe et al. (2003) showed that the aerobic power of octogenarian lifelong endurance athletes was approximately double that of untrained octogenarians (38 vs. 21 ml.kg^−1^.min^−1^). These remarkable aerobic capacities are the highest ever recorded in this age group. Some trained octogenarian athletes can still achieve high level of endurance performance. For example, the current marathon world record for a male octogenarian athlete was set in 2011 by a Canadian athlete (Ed Whitlock) who ran 3 h 15 min (http://www.world-masters-athletics.org/). This amazing performance corresponds to a running speed of 3.59 m.s^−1^ that is only 37% lower than that of the actual absolute world record (5.72 m.s^−1^, 2 h 02 min 57 s). Lepers et al. (2013c) have estimated that the VO_2max_ of Ed Whitlock was close to 50 ml.kg^−1^.min^−1^. In regards to Ed Whitlock's performance, we could expect in the future significant improvements in the performances of elderly master athletes who had high aerobic capacities when younger.

Reductions in the lactate threshold and exercise economy or locomotor efficiency in master endurance athletes may also contribute to the decrease in performance with advancing age but to a lesser extent compared to VO_2max_. The decrease in locomotor efficiency in master athletes remains controversial and may depend on the locomotion mode. For example, with cycling efficiency, an increase or a possible reduction (Sacchetti et al., 2010; Louis et al., 2012; Brisswalter et al., 2014) in efficiency with advanced age has been observed. The same inconsistent results have been reported concerning the energy cost of running in master athletes. For instance, Allen et al. (1985) did not observe a difference in running economy between young (25 years) and master athletes (56 years). In contrast, Sultana et al. (2012) reported a higher energy cost of running in master triathletes (≥40 years) when compared with their young counterparts. The higher energy cost of running in master athletes is associated with a lower muscle power. However, a long-lasting running exercise seems to preserve the spring-like mechanism (i.e., stiffness during running) of master athletes (Pantoja et al., 2016). The inconsistency of the results may be explained by the different age and fitness level of the tested master athletes. Interestingly, a recent study testing young (28 years) and master (60 years) triathletes showed that cycling efficiency was lower (−11%) and energy cost of running was greater (+11%) in the master compared with young triathletes (Peiffer et al., 2016); but when scaled to lean body mass, changes were more pronounced during the run (+22%). These findings suggest that at least within trained triathletes, aging can influence efficiency in both the run and cycle discipline. However, the effects of different types of training with advanced age, such as concurrent strength and endurance training (e.g., Louis et al., 2012; Piacentini et al., 2013) or polarized training (e.g., Pugliese et al., 2015) on exercise economy and more generally on endurance performance, remain to be explored.

**Figure 2:**
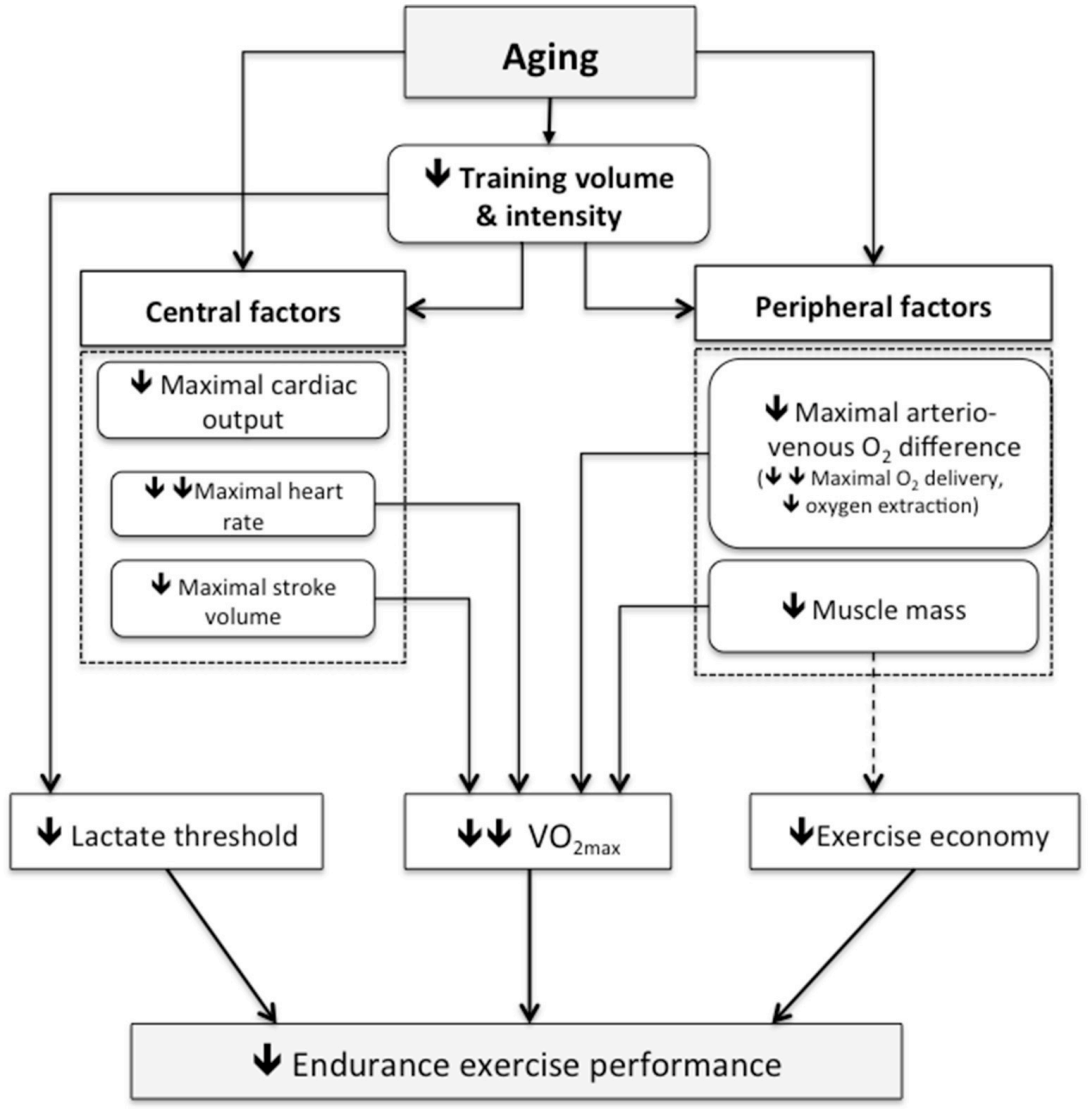
**Physiological mechanisms implicated in the age-related decline in endurance exercise performance**. A double arrow expresses a main influence.

The precise contribution of central (i.e., cardiovascular) and peripheral (i.e., oxygen extraction) factors to the reduced VO_2max_ with advancing age in master endurance athletes remains unclear (Figure 2). Both parameters of the Fick equation i.e., maximal cardiac output and maximal arterio-venous oxygen difference have been found to decrease in master athletes (Tanaka and Seals, 2008). The reduction in maximal cardiac output in master endurance athletes results from a reduction in both maximal heart rate and maximal stroke (Rivera et al., 1989; Ogawa et al., 1992). However, maximal heart rate seems to be the predominant mechanism mediating the reduction in maximal cardiac output with age. Interestingly, it seems that the rate of maximal heart rate decrease (~0.7 beat.min^−1^.year^−1^) with advancing age is similar between healthy sedentary, recreationally active and master athletes (Tanaka et al., 2001). At a peripheral level, maximal arterio-venous oxygen difference, which reflects in general the capacity of active skeletal muscles to extract and consume oxygen, has been found to decline modestly with age in master endurance athletes (Rivera et al., 1989). Moreover, it seems that maximal oxygen delivery, rather than oxygen extraction, is the major contributor to age-related decline in maximal arterio-venous oxygen difference in master endurance athletes (Tanaka and Seals, 2008). A decrease in maximal arterio-venous O_2_ difference may also be secondary to an age-related decline in of muscle mass, however chronic intense endurance exercise preserves muscle mass. For example, Wroblewski et al. (2011) showed that despite an increase in total body fat percentage with age, mid-thigh muscle area, quadriceps area, and specific strength did not decline significantly with age in master athletes. Dubé et al. (2016) recently showed that lifelong exercise was associated with higher intramyocellular triglyceride and glycogen content in all muscle fibers and higher metabolic efficiency during exercise. From a neuromuscular viewpoint, Power et al. (2010) also showed master runners preserved the number of functioning motor units in the tibialis anterior well into the seventh decade of life providing evidence that chronic activity has beneficial effects not only on the muscle fibers but also on the motoneuron. This same group of research also recently showed that world champion master athletes in their 9th decade of life had a greater number of surviving motor units, better neuromuscular transmission stability and a greater amount of excitable muscle mass as compared to age-matched controls (Power et al., 2016).

The inevitable age-related decline in endurance exercise performance of endurance master athletes is closely related with reductions in exercise training volume and intensity (Tanaka et al., 1997; Eskurza et al., 2002). However, the changes in the physical (e.g., prevalence of injuries, distribution of relative times spent at the different training intensities) and behavioral (e.g., reductions in time and motivation to train) characteristics of training of the endurance master athletes with age remain poorly described in the literature.

## Perspectives for future studies

Endurance exercise performance of master athletes continues to improve each year. In a descriptive point of view, we now need a model to compare the performance master athlete of different ages (Vanderburgh, 2015). We also lack information regarding training characteristics, nutrition habits, and recovery capacities of master endurance athletes (Louis et al., 2010; Brisswalter and Louis, 2014; Borges et al., 2016; Piacentini et al., 2016). Several other questions need to be addressed in the future, such as: Is endurance competition a greater psycho-physiological stressor for master athletes compared to young athletes (Piacentini et al., 2015)? Does response to high intensity interval training differ between young and old athletes? Is fatigue by induced prolonged endurance exercise amplified with advancing age? Are the benefits from chronic exercise observed in master athletes achievable in lifelong sedentary adults who begin exercising after age 40? We also need to identify the concomitant roles genetics and training in performance of master endurance athletes. Master athletes require the continued attention of sport scientists and exercise physiologists to extend their limits of (ultra)-endurance.

## Author contributions

All of the listed authors contributed to writing the manuscript (RL and PS), suggesting improvements to, and reviewing the manuscript (RL and PS). All the listed authors approved the final version of the manuscript.

### Conflict of interest statement

The authors declare that the research was conducted in the absence of any commercial or financial relationships that could be construed as a potential conflict of interest.
